# Proposed new definition for hospital-acquired SARS-CoV-2 infections: results of a confirmatory factor analysis

**DOI:** 10.1017/ash.2024.371

**Published:** 2024-09-09

**Authors:** Nicolás Reinoso Schiller, Claas Baier, Isabella Dresselhaus, Ulrike Loderstädt, Dirk Schlüter, Tim Eckmanns, Simone Scheithauer

**Affiliations:** 1 Institute of Infection Control and Infectious Diseases, University Medical Center Göttingen, University of Göttingen, Göttingen, Germany; 2 Institute for Medical Microbiology and Hospital Epidemiology, Hannover Medical School, Hannover, Germany; 3 Robert Koch Institute, Berlin, Germany

## Abstract

**Objective:**

The present study aims to develop and discuss an extension of hospital-acquired severe acute respiratory syndrome coronavirus 2 (SARS-CoV-2) infections (HA-SIs) definition which goes beyond the use of time parameters alone.

**Design:**

A confirmatory factor analysis was carried out to test a suitable definition for HA-SI.

**Setting and Patients:**

A two-center cohort study was carried out at two tertiary public hospitals in the German state of lower Saxony. The study involved a population of 366 laboratory-confirmed SARS-CoV-2-infected inpatients enrolled between March 2020 and August 2023.

**Results:**

The proposed model shows adequate fit indices (CFI.scaled = 0.959, RMSEA = 0.049). A descriptive comparison with existing classifications revealed strong features of our model, particularly its adaptability to specific regional outbreaks.

**Conclusion:**

The use of the regional incidence as a proxy variable to better define HA-SI cases represents a pragmatic and novel approach. The model aligns well with the latest scientific results in the literature. This work successfully unifies, within a single model, variables which the recent literature described as significant for the onset of HA-SI. Further potential improvements and adaptations of the model and its applications, such as automating the categorization process (in terms of hospital acquisition) or employing a comparable model for hospital-acquired influenza classification, are subjects open for discussion.

## Introduction

In the last two decades, there have major epidemic episodes related to viruses of the Corona family, including severe acute respiratory syndrome coronavirus 1 (SARS-CoV-1) affecting mainly Asia, Europe, and North America and severe acute respiratory syndrome coronavirus 2 (SARS-CoV-2) causing the so far biggest pandemic of the 21st century. Although the clinical presentations of these coronaviruses are mainly respiratory, there are, for instance, differences regarding the severity of the related diseases, the patient infectivity, and the incubation periods of each virus.^1^ In particular, knowledge of the incubation period is of great importance to draw appropriate conclusions from an infection prevention and control (IPC) point of view in the context of the public but also in hospitals.

Currently, most definitions for hospital-acquired SARS-CoV-2 infections (HA-SIs) use clear time limits between 7 and 14 days after admission to categorize a patient as having a HA-SI.^2,3^ However, the average length of stay of a patient in hospital is often much shorter than 14 days and even often does not exceed 7 days.^4,5^ Moreover, a 14 days limit seems impractical given that most cases present symptoms within 12 days of infection.^6^

Our understanding of nosocomial transmissions of SARS-CoV-2 still not ideal,^7^ for example, the incubation period or the period until an infected person becomes infectious for others may be shorter than initially thought^6^ with variations depending on the respective variant of SARS-CoV-2.^1,8^ Both the serial interval and infectivity demonstrate pronounced associations dependent on the variant^8^ making it difficult to define with certainty a nosocomial onset. This makes the task of classifying a nosocomial infection a problem, in which not only the time variable plays a fundamental role. Similar challenges may arise when employing initial symptoms as a primary criterion for defining nosocomial infections, owing to their variability, lack of specificity, and substantial overlap with other respiratory infections or preexisting pulmonary diseases.

Our study aims to develop and test a definition model based on simple and easily available routine surveillance data which addresses the probability of HA-SIs, beyond the solely use of time variables.

## Methods

### Study type and statistical analysis

A two-centered retrospective cohort study was conducted on 366 inpatients with a laboratory-confirmed SARS-CoV-2 infection. A confirmatory factor analysis was carried out to understand the role and factor structure of the proposed definition of HA-SI. Most of the observable variables (OVs) were coded in dichotomous terms (yes vs no), and therefore the data did not present a normal distribution. Given the dichotomous nature of the data, factor analysis employed the variance-adjusted weighted least squares (WLSMV) estimator for reliable results.^9,10^ Statistical analysis was conducted using R Studio version 1.4.1717 for macOS, with the “lavaan” package playing a key role.

### Data collection and data sample

Routine surveillance data were collected under the German Infection Protection Act at the University Hospital Goettingen and the Hannover Medical School in Lower Saxony, Germany.

All patients included in the study were inpatients with a positive nucleic acid amplification test (NAAT) at least 1 day after admission. Data were collected between 03/2020 and 08/2023, comprising 366 patients (146 women; 39.9%; Table 1) with laboratory-confirmed SARS-CoV-2 infection. Patients with a positive NAAT immediately before or within the day of admission as well as known SARS-CoV-2 positive inpatients transferred from other hospitals, and all outpatients were not considered.

**Table 1.** Sample characteristics

	Male (*N* = 220)	Female (*N* = 146)
Age in years	61.45 ± 19.58	55.5 ± 24.19
Mean duration in days between hospital admission and initial positive SARS-CoV-2 test	10.83 ± 20.21	8.43 ± 13.57

### Variables selection

The selection of variables was based on IPC experience, current contact tracing, and management insights as well as on published literature.

The decision to select as separation criteria between the “Community,” “Indeterminate,” and “Highly probable nosocomial” (HPN) categories the days 4 and 10 after hospital admission has been prompted by the results of Lauer et al. (2020), which indicate that the median of infected persons present symptoms within the first 5 days of infection.^6^ Nonetheless, recent data suggest a shorter incubation period for the virus variants delta (4.3 days after infection) and omicron (3.6 days after infection).^1^

We also chose the variable “Regional incidence” (RI) for the date of the patient’s first positive NAAT. Both the regional incidence and the variable “Strong indication that a positive healthcare worker or patient was involved” were included in the model based on the results of two publications, the first by the German Robert Koch Institute indicating a higher probability of outbreaks when the regional incidence is higher.^11^ Second, the significant role of healthcare worker transmissions in mediating HA-SI is evident throughout the pandemic context and has been demonstrated on multiple occasions.^12,13^

The variable “Cluster (high possibility of an outbreak)” was scored as present if there were at least 2 other SARS-CoV-2 cases in a 4-day period around the detection at the respective ward.

To avoid errors, we have decided to use the symptom variables “Early onset of symptoms after admission” for the first 3 days mainly because reporting of the mild symptoms are more rigorous in that period than later, where the exact time of symptom onset is often difficult to determine or unclear.^14^ Rather than the exact time of symptom onset, the variables indicate whether the patient has had symptoms related to a SARS-CoV-2 infection within these days.

The variable “Days since the last hospitalization” was introduced to especially address the factor of a new inpatient admission shortly after hospital discharge. This variable was considered positive, if patients were discharged within the last 3 days prior to readmission and were hospitalized for at least 4 days in that previous stay.

The variable “Screening” indicates whether a SARS-CoV-2 test was performed on admission regardless of symptoms or anamnesis and “Early physician estimation” involves clinicians categorizing the transmission source as community-acquired or indeterminate based on their clinical expertise, even with a negative admission test.

### Model development

Our model definition was developed by experts within the project “Preparedness and pandemic response in Germany” (PREPARED) using previously discussed literature (see variable selection). It integrated factors that were available or easily obtainable by any hospital and discarded factors that were not systematically collected for patients during their hospital stay. The model uses nine factors (OV) divided into three categories or latent variables (LV), with a subject to item ratio suitable for the analysis.^15^

Once the operationalization displayed, the LVs to be measured are formed as mentioned in Table 2.

**Table 2.** Variables incorporated in the PREPARED model

Latent variable	Observable variables
Community-acquired	Positive NAAT result within 3 days after admission
	Screening
	Early onset of symptoms after admission
	Early physician estimation
Indeterminate	No symptoms of SARS-CoV-2 infection on admission AND first positive NAAT result 4 to 9 days after admission
	Days since the last hospitalization
	Early physician estimation
Highly probable nosocomial	Days since the last hospitalization
	Regional incidence
	Strong indication that a positive healthcare worker or patient was involved
	Cluster (high possibility of an outbreak)

To perform a comprehensive comparative analysis between the results obtained from the PREPARED model and the official European Center for Disease Prevention and Control (ECDC) definition from 2021^16^ (Appendix 1). We consolidated the ECDC categories of “probable nosocomial” and “definite nosocomial” into a single category. This merger facilitated a descriptive comparison.

## Results

**Figure 1.**
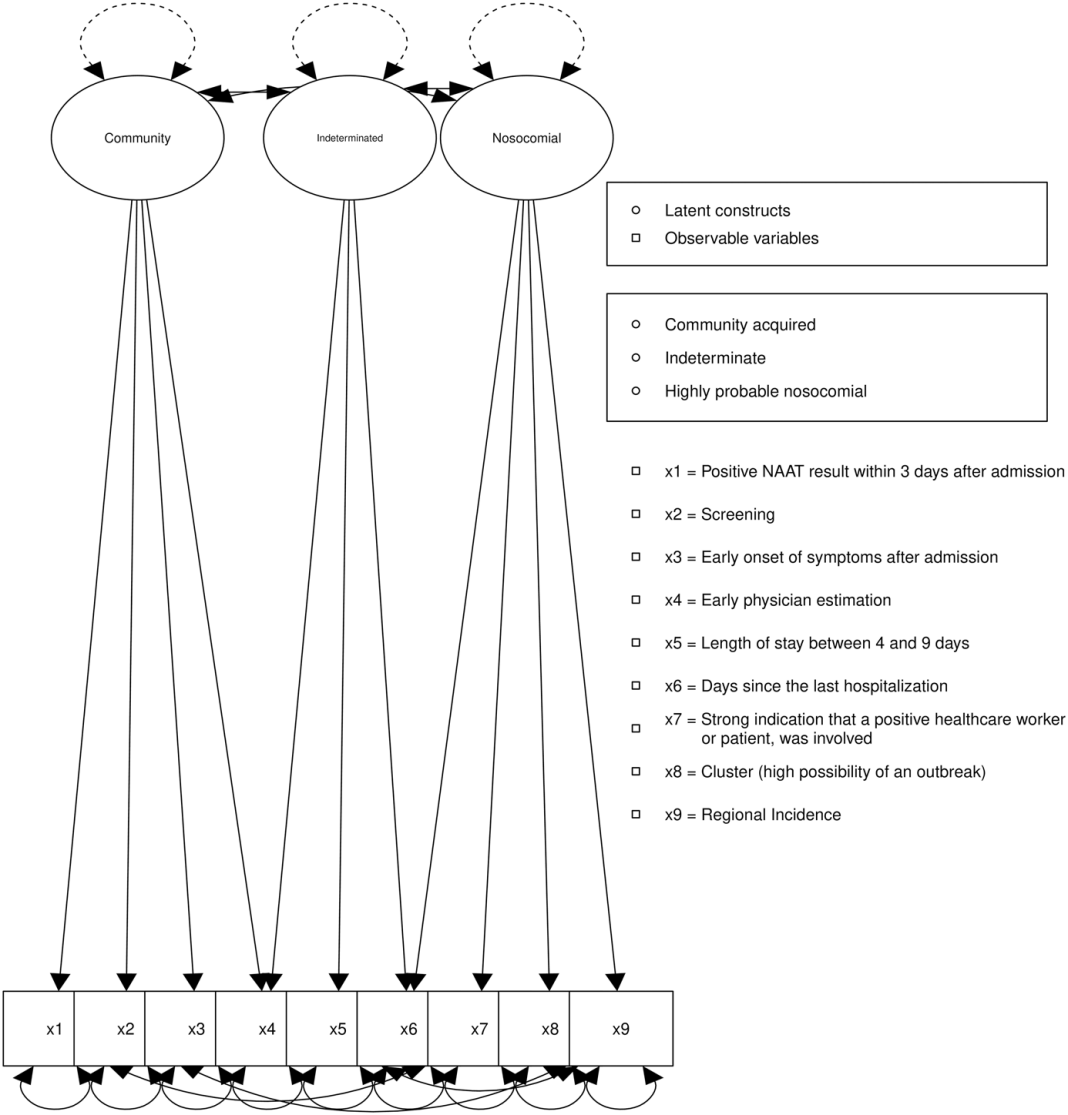
PREPARED structural equation model.

The model presented in this study (Figure 1) exhibits a satisfactory comparative fit index (CFI) as outlined in Table 3. However, we decided to conduct an analysis of modification indices to better define the model.

**Table 3.** CFA fit indices

Model	CFI	CFI.scaled	RMSEA	RMSEA.scaled	SRMR	Estimator
PREPARED model	0.954	0.876	0.084	0.109	0.064	WLSMV
PREPARED model after modifications	0.988	0.959	0.049	0.067	0.042	WLSMV

* CFI: comparative fit index

* RMSEA: root mean square error of approximation

* SRMR: standardized root mean square residual

Modification indices revealed specific areas for potential model refinement. Plausible improvements were carefully evaluated and incorporated into the model if supported by theoretical justifications. We allowed the following modifications:

- The positive covariance between the observed variables RI and “early onset of symptoms after admission” had a significant impact on the model, leading to an increase in the model fit.
- The positive covariance between the observed variables RI and “positivity between the 4th and 9th day after admission” significantly improved the model fit.
- The negative covariance between “positive NAAT result within 3 days after admission” and “cluster (high possibility of an outbreak)” pointed to a negative association, contributing to an improved model fit.

The modifications significantly influence the model’s goodness of fit (ΔCFI > 0.05) and have been incorporated into its latest version. The resulting model demonstrates improved robust fit indices and displays moderate factor loadings between the LVs and OVs. There are two negative correlated factor loadings corresponding to the OV “Screening” and an infection in the context of Community and to the OV “Days since hospitalization” with the LV “indeterminate,” both results in line with the theoretical implications to be discussed below. The factor loadings of the model oscillate between moderate and low values. However, the correlations between the LV “indeterminate” with the LV “Community” (−0.827) and with “HPN” (0.677) are within an acceptable range. A strong negative correlation (−0.942) was displayed between the LVs “Community” and “HPN.” These correlations will be addressed during the discussion together with some possible explanations for the displayed factor loading of the model.

When analyzing the distribution of cases between the ECDC and PREPARED categorizations, it emerges that the PREPARED categorization assigns fewer undetermined cases. These cases are distributed between the community and HPN categories, as shown in Table 4. Specifically, the PREPARED model demonstrates a 10% reduction in total indeterminate cases compared to the ECDC categorization. Moreover, in 2020, the PREPARED categorization identified 17% of cases as HPN, compared to 22% categorized by the ECDC model. The disparities in case distribution across both categorizations are detailed in Table 4 and can be observed in Appendix 2.

**Table 4.** Case numbers per year across the PREPARED and EDCD categorizations

Years	2020 (*N* = 41)	2021 (*N* = 135)	2022 (*N* = 110)	2023 (*N* = 80)	Overall (*N* = 366)
**PREPARED**					
Community	26 (63.4%)	68 (50.4%)	18 (16.4%)	63 (78.8%)	175 (47.8%)
Indeterminate	8 (19.5%)	26 (19.3%)	16 (14.5%)	2 (2.5%)	52 (14.2%)
Highly probable nosocomial	7 (17.1%)	41 (30.4%)	76 (69.1%)	15 (18.8%)	139 (38.0%)
**ECDC**					
Community	23 (56.1%)	56 (41.5%)	13 (11.8%)	60 (75.0%)	152 (41.5%)
Indeterminate	9 (22.0%)	42 (31.1%)	29 (26.4%)	8 (10.0%)	88 (24.0%)
Highly probable nosocomial	9 (22.0%)	37 (27.4%)	68 (61.8%)	12 (15.0%)	126 (34.4%)

## Discussion

This study successfully unifies, within a single model, variables which the recent literature identified as significant for the HA-SIs, while managing to adequately identify the risk of nosocomial cases.

The use of prevalence and incidence models to determine the risk of infection has been used for instance to determine the risk of HIV infection through blood transfusions.^17,18^ Due to the nature of the use case at issue, we do not speak of a prevalence–incidence model but instead use incidence as a key variable of the model; in other words, we assume a near to zero prevalence of SARS-CoV-2 within hospitals. Among the advantages of using regional incidence as a factor is the relatively simple and rapid use of it to determine the likelihood risk of nosocomial infection, similar to the incidence rate/window period model.^18^ A prerequisite for this approach is, of course, that there are reliable and representative figures for the regional incidence. This also depends on the local testing strategies.

Overall, the model presents an adequate fit, indicating that the concepts defined by the OV are plausible and can be helpful in categorizing patients infected with SARS-CoV-2. However, further examination of the model showed that there is a high correlation between the “Indeterminate” and “HPN” category. This correlation raises two interesting facts. The first one is that we can conclusively separate patients with a clear non-hospital-acquired infection from the rest. Data-based support in assessing whether a SARS-CoV-2 infection that first occurs in hospital is truly nosocomial or whether it is brought along from the community (in the incubation phase) is very valuable from an IPC perspective. In practice, relevant hygienic measures depend on this assessment. The second one is that there is a need for a better characterization of “Indeterminate” patients. This is undoubtedly a difficult task that has not been a focus of this study but could represent an improvement of the model while at the same time enabling a better distinction between the categories.

Analyzing the results in detail, it can be seen that there is a negative loading factor between the LV “Community” and the OV “Screening” (−0.258), indicating that a negative screening result correlates inversely with community-acquired infections, which highlights the importance of screening to better determine cases of HA-SI adequately. Given the current circumstances, this negative link corroborates existing literature.^3^ Furthermore, it could be advisable to conduct screenings in hospitals during an specific epidemiological context such as seasonal incidence peaks or outbreaks using antigen rapid tests rather than NAAT, to achieve similar results.^19–22^ Overall, these results could aid in adjusting testing strategies to identify patients with clear HA-SI using straightforward variables, significantly enhancing patient safety and reducing additional costs and workload.

The modifications applied to the model, specifically the inclusion of covariances between regional incidence and “Early onset of symptoms after admission,” are theoretically grounded. The epidemiological context (displayed by the local incidence rates) undeniably shapes the early onset of symptoms after hospital admission. The model extension, which allows the covariance between RI and “positive cases between the 4th and 9th days after admission” is supported by both empirical evidence and theoretical understanding. A simulation study by Smith et al. (2022) suggested that the higher the regional incidence, the less effective is screening in preventing transmission.^21^ Furthermore, a study using whole-genome sequencing in France displayed an association between the regional incidence and HA-SI cases.^23^ The final modification to the model, introducing covariances between “Positive PCR result within 3 days after admission” and “Cluster (high possibility of an outbreak),” reveals a negative association. The theoretical foundation for this association is rooted in the likelihood of being part of a cluster or outbreak; it suggests that testing positive within the first 3 days after admission should be less likely than after this initial period. This covariance is not only plausible but also enhances the model significantly, leading to its incorporation and acceptance.

The PREPARED model appears better at distinguishing HPN from community cases than the ECDEC definition, reducing the numbers of indeterminate cases. The PREPARED model compared with the ECDC classification displays fewer numbers of HPN during the year 2020. However, it shows 8% more cases in HPN cases in 2022 within the same data set, suggesting a potential link to the onset of the Omicron variant, which led to more local and hospital outbreaks and a significant increase in regional incidence rates.^24^ A distinctive feature setting the PREPARED definition apart from others is its integration of regional incidence rates. This unique characteristic imparts a significant level of resilience against fluctuations in variants of concern. Moreover, it could potentially account for the disparities observed in the distribution of HPN and indeterminate cases between both categorizations. Its adaptability to specific regions and prevailing conditions within a given epidemiological context stands as a testament to its robustness and practicality for future outbreaks.

The shift of almost 10% of the cases from indeterminate to the other categorizations has interesting implications for clinicians and IPC experts. From an IPC standpoint, this shift could significantly contribute to refining the definition of outbreaks and subsequently enhance the analysis of their sources. Furthermore, moving cases out of the indeterminate category may facilitate the identification of more specific risk factors for nosocomial transmissions of SARS-CoV-2. It also opens the possibility of reassessing the overall length of stay for nosocomial patients and better understanding the burden imposed by these infections.

The improved differentiation between nosocomial and non-nosocomial patients holds the potential for enhancing prevention strategies. In regions with high incidence rates, clinicians can use this distinction to tailor their approaches accordingly. This shift in categorization may prompt clinicians to consider regional incidence rates more carefully, thereby optimizing prevention methods and fostering more effective healthcare practices.

The PREPARED definition also presents an opportunity to use routine data to automatize the classification process leaving indeterminate cases for the physician or IPC evaluation.

Although this study provides valuable insights, it is important to acknowledge its limitations. The argument for performing a Confirmatory Factor Analysis (CFA) without prior Exploratory Factor Analysis was the sample size^25^ as well as possible model overfitting.^26^ In addition, the quality of the study is affected by the SARS-CoV-2 variants predominant at the time of data collection. Although the data were obtained by two university hospitals, both located within the same German federal state. Possible variables could broaden the definitions of HA-SI and have not been incorporated into the model due to lack of data. This applies for instance to genomic data which was not widely available. However, genomic data could constitute a major advantage to better confirm or exclude nosocomial transmission. In fact, sequencing has previously been used to determine outbreaks and nosocomial infections very effectively for different kinds of pathogens.^27^ Additionally, variables such as medical history or ethnicity were not included due to insufficient data. This may vary in other regions and may be a valuable addition to the model in the future. As for age and gender, in the previous analysis, they did not have an impact on the nosocomial incidence, but rather on disease severity or mortality. Similarly, vaccination status was not included due to data protection regulations, which could affect the sensitivity of the model and is perhaps its most significant limitation. Although the model is effective and illustrates a novel implementation of CFA to test nosocomial definitions, it could benefit in the future from the availability of variables such as genomic data or vaccination status.

In summary, this study presents a new model for the definition HA-SIs. By leveraging readily available data typically accessible to physicians and IPC experts, this definition enables a swift and relatively straightforward assessment of patient infection sources.

## Appendix A

European Center for Disease Prevention and Control (ECDC) definition

**Table:**

**Community-acquired infection**	Symptoms or sample present on admission or with onset on day 1 or 2 after admission (or on days 3–7 with a strong suspicion of community transmission)
**Indeterminate healthcare-associated infection (HAI):**	Symptom onset or sample present on days 3–7 after admission with insufficient information on the source of infection to assign to another category
**Probable HAI**	Symptom onset or sample present on days 8–14 after admission (or on days 3–7 and a strong suspicion of healthcare transmission)
**Definite HAI**	Symptom onset or sample present on day > 14 after admission
